# Reconstruction of anterior chest wall: a clinical analysis

**DOI:** 10.1186/s13019-018-0810-x

**Published:** 2018-12-07

**Authors:** Erji Gao, Yang Li, Tiancheng Zhao, Xiang Guo, Weiwei He, Weiming Wu, Yonghong Zhao, Yi Yang

**Affiliations:** Department of thoracic surgery, Shanghai Jiao Tong University Affiliated Sixth People’s Hospital, Shanghai, 200233 China

**Keywords:** Sternal tumor, Titanium sternal fixation system, Reconstruction of the chest wall

## Abstract

**Objective:**

To investigate the methods and clinical efficacy of reconstruction of chest defects with titanium sternal fixation system after the surgical resection of sternal tumors.

**Methods:**

A total of 6 patients with sternal tumor who were diagnosed and underwent resection and repair of the chest wall defects by titanium plates system, from 2017.3 to 2017.11 in our hospital were reviewed. Their pathological types, surgical reconstruction methods, follow-up results were analyzed.

**Results:**

Six cases of sternal tumor were completely resected and the sternums were reconstructed with titanium sternal fixation system. There was no operative death, postoperative chest wall deformity, abnormal breathing or complications of respiratory circulation. After 3 to 10 months of follow-up, there was no loose screw or plate exposure. Not only the thoracic appearances were good, but patients’ satisfaction was high.

**Conclusions:**

Surgical resection is the best treatment for sternal tumors, no matter it is benign or malignant. Titanium sternal fixation system combine with other soft materials can reconstruct the chest wall well after resection, and this technique is efficient as well as easy to learn.

## Background

Sternal tumors are relatively rare, and there are few reports in the literature. Surgical resection is the main treatment. However, when the sternal defect is large, the respiratory function may be seriously affected. Then, thoracic reconstruction is required. This is the key and difficult procedure of surgical treatment. Although there are methods such as 3D printing materials, ordinary metal and metal mesh, they either are complicated to operate or have limited availability so they cannot be clinically promoted.

## Methods

We select 6 representative cases of sternal tumors whose chest wall was reconstructed with titanium sternal fixation system in our hospital from March 2017 to November 2017 to share our experience in reconstructing different kinds of anterior chest wall defects. After a maximum follow-up of 10 months, we evaluated our operative results depend on chest films and their degree of satisfaction about the postoperative motor function and appearance. They are reported below.

All patients were hospitalized for finding a lump or chest pain for some time in anterior chest wall. But case 5 had a medical history of bilateral thyroidectomy 3 years ago and sternal destruction was found before the operation. After thyroidectomy, radiotherapy and isotope therapy were applied for the sternum mass. More information is showed in Table 1.

**Table 1:** clinical characteristics of all cases

case	Age (year)	gender	work	Location of tumur	Size of defect(cm)	Time of return to daily work(week)	degree of Satisfaction
1	60	male	Retire	manubrium	10*12	4	8
2	39	male	driver	sternoclavicular joint	12*8	2	9
3	49	male	clerk	mesosternum	7*8	2	10
4	45	female	teacher	mesosternum	6*8	3	9
5	64	male	Retire	mesosternum	12*8	8	6
6	38	male	freelance	mesosternum	10*10	3	8

### Operative techniques

1. Tumor resection

All patients received a single lumen endotracheal tube with backside elevated in the supine position and upper limbs abduction. The chest wall was disinfected routinely and the surgical incision was on the basis of tumor location. All surgeries were finished with a median sternotomy in this series except for case 1 with a bilateral supraclavicular and median sternum united T-shaped incision and case 2 with a right supraclavicular and median sternum united 7-shaped incision. Every patient had a wide resection by cutting bilateral ribs and sternum 2–3 cm away from the edge of neoplasm, but tried to reserve manubrium to the greatest extent.

2. Chest wall reconstruction

**Fig. 1:**
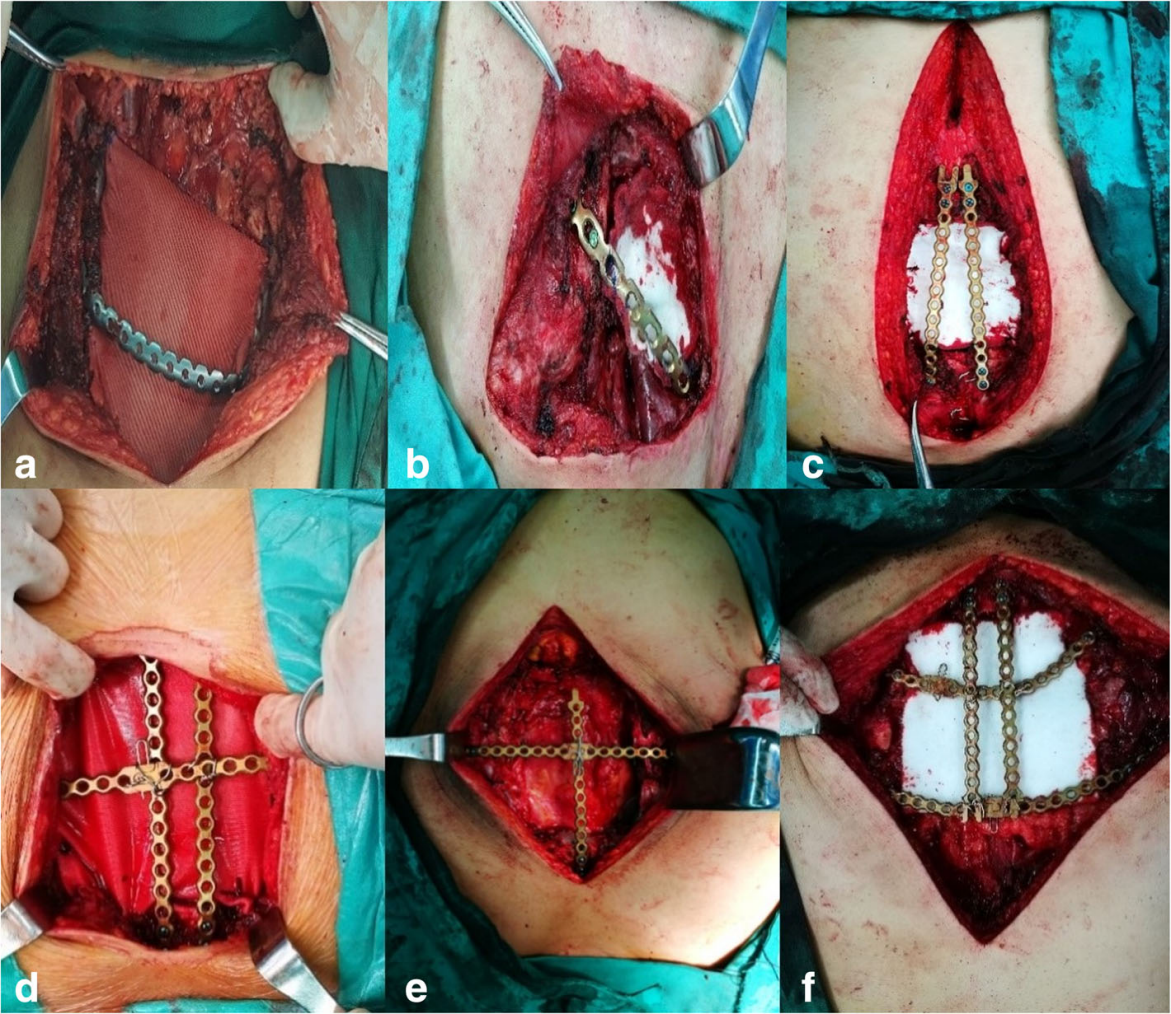
Intraoperative view of technique of the bars fixed with multiple screws and polyester fabrics sutured beneath it. (**a** is for Case 1, **b** is for Case 2, **c** is for Case 3, **d** is for Case 4, **e** is for Case 5, **f** is for Case 6)

Firstly, our techniques change from the location and size of sternal defects. (1) For those patients whose manubrium had been resected, we got 1 bar or 2 from titanium sternal fixation system according to the size of defect arched across the defect and threated the bar to bilateral ribs like Case 1(Fig. 1a). Another case was that the manubrium had been partly resected, we just need to threat the bar one side to rib and another to sternum like Case 2(Fig. 1b). There was no need to get a bar fixed vertically in these patients. (2) For the patients whose manubrium had been reserved, we needed 1 bar or 2 according to the size of defect to be threated to manubrium vertically first of all. When the distal part of remaining sternum was adequate for screws to block the bar then just fixed the distal part of the vertical bar to it like Case 3(Fig. 1c). If the distal part of remaining sternum was inadequate for screws to block the bar then another bar was needed to arch across the defect and fix it to bilateral ribs and bundle it with the vertical one at the same time like Case 4(Fig. 1d) and 5(Fig. 1e). Another case was no distal part of sternum left then 1 bar or 2 according to the size of defect was needed to arch across the defect and fix it to bilateral ribs and also bundle it with the vertical one like Case 6(Fig. 1f). Secondly, we used polyester fabrics to reconstruct soft tissue defect. Lastly, a mediastina drainage tube, a pressure bandage of reconstruction area and a chest strap fixation were necessary.

## Results

**Fig. 2:**
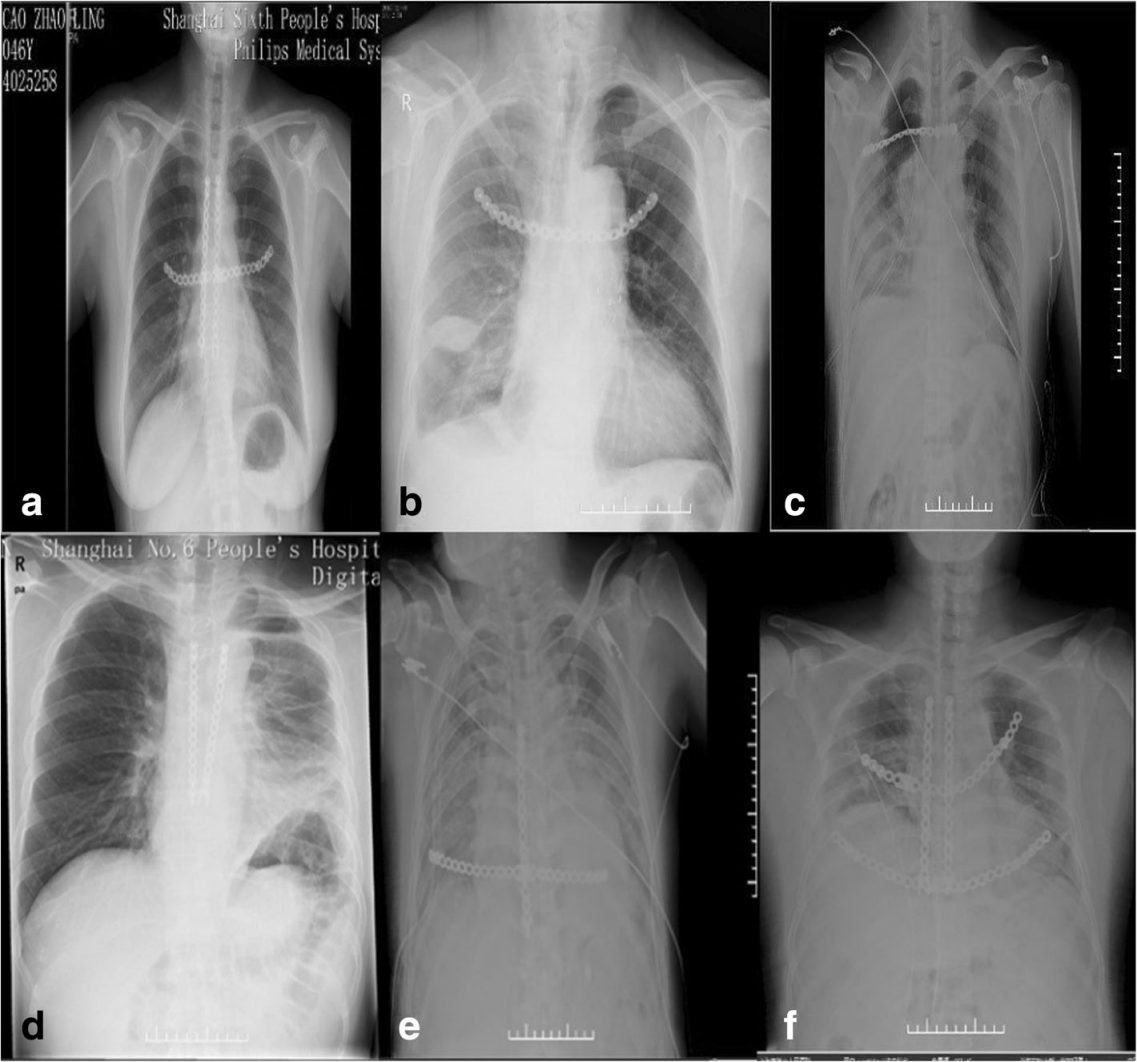
Postoperative X-ray films of the case series. (**a** is for Case 1, **b** is for Case 2, **c** is for Case 3, **d** is for Case 4, **e** is for Case 5, **f** is for Case 6)

The pathological diagnoses of this series were the sternal metastasis of follicular thyroid carcinoma, cancerization of osteochondroma (peripheral chondrosarcoma), classic Hodgkin’s disease (nodular sclerosis type), giant cell tumor of bone, sternal metastasis of thyroid cancer and chondrosarcoma in order. All patients had a perfect postoperative recovery without complications except for the fifth case there was little exudation from whose incision but quickly healed by intensified anti infection and dressing change. Every patient got an X-ray film exam before leaving hospital and it showed that the location of internal fixation and the cosmetic appearance was wonderful (Fig. 2). During the follow-up, we evaluated our operative result depend on chest films and the patients’ degree of satisfaction about the postoperative motor function and appearance. We asked patients to grade the treatments according to postoperative motor function and appearance. The score ranged from 0 to 10, and 0 means they were very disappointed, on the other hand, 10 means they were very satisfied. We can see they were satisfied with the results on a different level from Table 1.

## Discussion

The optimal therapy for sternal neoplasm is surgical resection and most surgeons agree that the resection should be at least 2 cm away from the sternal neoplasm margin. But the resection of sternal neoplasm may result in huge anterior chest wall defect that would break the integrity of chest wall and lead to severe affection of respiration and circulation. Therefore, it is necessary to reconstruct the huge chest wall defect result from resection of sternal tumor [1].

Most surgeons agree that defects of chest wall > 5 cm in diameter should be reconstructed particularly for those full-thickness or anterolateral defects [2]. As far as we know, an ideal material and technique of reconstruction shall meet the following requirements: (1) high rigidity and stability to avoid chest wall floating; (2) keep in body for long time without rejection reaction and likelihood of infection; (3) easy to cut, shape and fix; (4) be convenient for sterilization; and (5) radiolucency to make an anatomic reference to do a better follow up and identify a possible local neoplastic relapse [3]. Although there are many methods for the reconstruction of sternal defect, include the reconstruction of soft tissue by free flaps, greater omentum and polyester patch. And the restore of chest wall rigidity with three-dimensional printing bioscaffold [4], metal plate or mesh, polypropylene mesh [5] and allograft or homograft of bone. But none have proven to be clearly superior. Muscle flaps, omentum and bone grafts are able to incorporate into native tissue with revascularization and cellular repopulation, making them more resistant to infection. On the other hand, the amount of that often be limited, the surgical procedures are more complicated and more likely accompanied by additional operation trauma. Titanium matches to the hardness and elasticity modulus of skeleton and has a high resistance to erosion, a low density, more than that, it is highly biocompatible. The titanium plates are easy to model and fix, they all contribute to a higher thoracic stability and better cosmetic result [6].

## Conclusions

The titanium plates system we used in this series is a new generation of rib prosthesis system called Titanium Sternal Fixation System. Each plate contains two symmetric parts jointed by a plug that can be taken away with ease for adjusting fixation position during operation. But, above all, plates of this system have holes for screws to threaten them to the ribs instead of stabilizing trough friction between the plate and bone generated by compression like other unlocking plates so that the periosteal blood supply and rib perfusion are preserved. The combination of titanium plates system and polyester patch we used to reconstruct thoracic wall defect has many advantages, on the other hand, avoided the great disadvantage of deformation, chronic pain, fading of tension and paradoxical respiration that may occur in patient whose defect is only reconstructed with meshes, or infection, poor elasticity, plate fracture/dislocation and paradoxical respiration. Moreover, the titanium plates system is simple and need less time in operation procedure. All patents had a cosmetic appearance without any respiratory distress and the midsternal organs are well protected. After our longest 10 months’ clinical follow up, there are no complications occurred in this case series. Last but not least, our method of reconstruction with titanium plates is suitable for any size of defects not like the custom-made titanium plate [7]. We recognize this is just a primary study and need much further research inevitably in the future.
